# Motion Artifact Correction of Multi-Measured Functional Near-Infrared Spectroscopy Signals Based on Signal Reconstruction Using an Artificial Neural Network †

**DOI:** 10.3390/s18092957

**Published:** 2018-09-05

**Authors:** Gihyoun Lee, Sang Hyeon Jin, Jinung An

**Affiliations:** Convergence Research Center for Wellness, DGIST, Daegu 42988, Korea; ghlee@dgist.ac.kr (G.L.); jinjinsh@dgist.ac.kr (S.H.J.)

**Keywords:** functional near-infrared spectroscopy, motion artifact, artificial neural network, signal entropy, wavelet transform

## Abstract

In this paper, a new motion artifact correction method is proposed based on multi-channel functional near-infrared spectroscopy (fNIRS) signals. Recently, wavelet transform and hemodynamic response function-based algorithms were proposed as methods of denoising and detrending fNIRS signals. However, these techniques cannot achieve impressive performance in the experimental environment with lots of movement such as gait and rehabilitation tasks because hemodynamic responses have features similar to those of motion artifacts. Moreover, it is difficult to correct motion artifacts in multi-measured fNIRS systems, which have multiple channels and different noise features in each channel. Thus, a new motion artifact correction method for multi-measured fNIRS is proposed in this study, which includes a decision algorithm to determine the most contaminated fNIRS channel based on entropy and a reconstruction algorithm to correct motion artifacts by using a wavelet-decomposed back-propagation neural network. The experimental data was achieved from six subjects and the results were analyzed in comparing conventional algorithms such as HRF smoothing, wavelet denoising, and wavelet MDL. The performance of the proposed method was proven experimentally using the graphical results of the corrected fNIRS signal, CNR that is a performance evaluation index, and the brain activation map.

## 1. Introduction

Near-infrared (NIR) light can be absorbed differently by various biological chromophores in the human brain. In particular, the absorption spectra of oxy-hemoglobin (HbO) and deoxy-hemoglobin (HbR) are distinct at NIR wavelengths between 650 nm and 950 nm [1]. Functional NIR spectroscopy (fNIRS), which can be employed to measure regional changes in the concentrations of HbO and HbR by using a noninvasive optical neuro-sensor called an NIR optode, is a method of noninvasively monitoring regional cerebral blood flow variations and brain activity [2,3]. fNIRS provides results similar to the blood-oxygenation-level-dependent (BOLD) outputs of functional magnetic resonance imaging (fMRI) for cortical regions. fNIRS has important advantages over the other neuroimaging modalities such as fMRI, positron emission tomography, electroencephalography, and magnetoencephalography; these advantages include low cost, portability, and the potential also to be applied in various ecological environments [4]. As fNIRS is a relatively new modality compared to the conventional neural modalities, it still does not have a universally accepted data analysis framework.

The general linear model (GLM) [5] explains data as a linear combination and is the standard method of analyzing fMRI data. Numerous statistical analysis toolboxes for fNIRS based on the GLM have been developed [3,6,7,8,9,10]. However, the GLM can fail when there is noise in the NIRS measurements. This noise can arise for various reasons, such as subject movement, blood pressure variation, and instrumental instability [11,12]. As an NIR light source is employed in fNIRS, this method is free of electrical artifacts and is fairly robust against motion artifacts. However, when a subject moves, the fNIRS optodes may shift relative to the head and alter the coupling between the optodes and scalp. Thus, the artifacts caused by head motion are among the most important factors limiting the quality of optical data in neuroimaging applications [13]. Motion artifacts can easily be confused with actual hemodynamic responses due to brain activity and are characterized by abrupt changes, thereby potentially resulting in spikes with amplitudes much larger than that of the true signal [14].

Recently, numerous noise correction algorithms have been developed for fNIRS. In previous studies, frequency band-pass filter-, moving average-, and Wiener filter-based algorithms [13] enabled the effective elimination of measured high-frequency noise and low-frequency signal drift [6,15,16,17]. However, the frequency filter-based algorithms often failed to remove noise with frequency features similar to those of the hemodynamic response, such as head motion artifacts. Adaptive filters based on Wiener filters have been widely used for noise reduction in various biomedical applications. However, such filters require additional reference signals and have usually failed to remove abrupt noise. The discrete cosine transform (DCT) [18] has been widely used as an efficient high-pass filter. Because the DCT does not consider hemodynamic characteristics, it cannot achieve outstanding performance when applied to fNIRS signals. A smoothing technique based on the hemodynamic response function (HRF) [19] was also proposed, which is a very powerful low-pass filtering method in which the hemodynamic characteristics are considered based on the hemodynamic response of the brain. However, the HRF smoothing method cannot correct low-frequency artifacts, such as the baseline drift of an fNIRS signal, because it mainly performs low-pass filtering. Lately, some studies have been conducted on wavelet approaches for fNIRS detrending. Wavelet transformation (WT) has been widely applied for signal denoising, compression, detection, and pattern recognition. It has also been adapted for fNIRS signal analysis, including detrending and classification [20,21]. The wavelet minimum description length (MDL) method, which is a wavelet-based fNIRS detrending algorithm [11], has proven to be quite effective. However, it can lead to hemodynamic response attenuation in specific experimental situations, such as tasks involving rapid motion and one-block tasks. In general, multiple channels are employed in fNIRS to cover a wide brain cortex area. Each channel has different NIR data characteristics and noise features because of skin, hairs, emitters, detectors, optical cables, and so on at each location. In addition, some channels are affected by motion artifacts, while others are not and therefore do not require any motion artifact correction. If the same correction method is applied to each channel, signal distortion or errors may occur. Therefore, it is essential to perform motion artifact correction separately for each measurement location.

The decision-making procedure of the ANN is based on the features of the input patterns and has been widely applied for biomedical data classification [22]. The classical feed-forward neural network (FFNN) structure has one input layer, one output layer, and a few hidden layers with hidden neurons. The connections of the neurons in the different layers are supplied by adjusting the weights, and each neuron is connected to the neurons in the next layer [23]. The input signal needs to spread forward from the input layer to the neurons in the hidden layers and is transformed by the activation function. Then, the input signal is transmitted from the neurons in the hidden layers to the output layer [22]. The FFNN can be trained as a machine leaning technique that uses the generalized back propagation algorithm (BPA), which is a supervised learning algorithm.

In this paper, we suggest a new motion artifact correction method involving an entropy-based most contaminated channel identification algorithm and motion artifact correction with an artificial neural network (ANN). The most contaminated channel identification algorithm is employed to determine the most contaminated fNIRS optode and channel, which are characterized by unbalanced indices computed by performing entropy cross-correlation with the neighbor channels around each fNIRS optode. Then, the contaminated fNIRS channel is reconstructed by using a back-propagation neural network (BPNN) that is trained to prefer the signals through the normal neighbor channels. The experimental results show that the proposed method outperforms the conventional approaches.

## 2. Methods

### 2.1. fNIRS Data Acquisition

**Figure 1:**
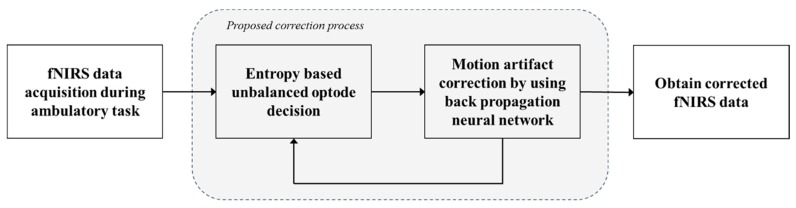
Block diagram of the proposed method.

The analysis of functional hemodynamic changes in neuroimaging methods such as fNIRS and fMRI is generally based on an assumption of linear addition of hemodynamic changes [24]. Figure 1 shows a block diagram of the proposed motion artifact correction method. Each step will be described in this session. The modified Beer-Lambert law (MBLL) [25] describes optical attenuation in a highly scattering medium such as human tissue, and the optical density variation of the HbO concentration changes (ΔcHbO) can be described using the MBLL [6]. The first step is raw fNIRS data acquisition by using a modified GLM. The GLM has been widely used as a standard method of fNIRS signal analysis, and it can be adopted for the interpolated measurement of ΔcHbO. Friston et al. showed that a BOLD signal can be approximated using a convolution model between a stimulus function and the HRF, in which the time-dependent sensorimotor or cognitive parameter of interest was similarly modeled [26]. There are many possible models for the HRF. The canonical HRF, which is composed of two gamma functions, was employed in Reference [27]. Additionally, the derivatives of the HRF with respect to delay and dispersion can be used to mitigate the problem that the precise shape of the HRF varies across the brain [27]. Adaptive estimation of the HRF using multiple gamma functions can also be used in fNIRS to account for oxygen-species-dependent hemodynamics variations [6].

**Figure 2:**
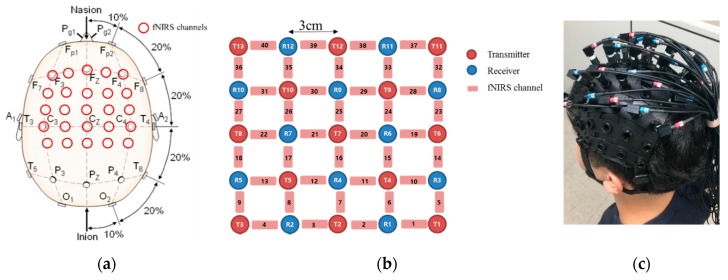
fNIRS channel information: (**a**) channel locations on the brain image; (**b**) schematic diagram of the T- and R-optodes; (**c**) fNIRS cap with 40 wired channels.

An fNIRS brain imaging system (FOIRE-3000, Shimadzu, Japan) was used for data acquisition. This instrument sends multiple wavelengths of laser light (780, 805, and 830 nm) from each T-optode to each R-optode and it has an fNIRS data sampling rate of 10 Hz. The arrangements of the channels and optodes are shown in Figure 2. Figure 2a depicts the channel locations in the brain image, and Figure 2b presents the 5 × 5 structure of the optodes forming the fNIRS system with 40 channels, 13 transmitting (T) optodes and 12 receiving (R) optodes. The distance of neighbor optode is 3 cm and the T- and R-optodes can cover the motor and sensory regions of the cortex. The fNIRS channel information and neighbor channels of each optode can be identified using Figure 2. Neighbor channels are those that are measured from the same optode. Figure 2 shows that the fNIRS channels can almost cover the supplementary motor area and primary motor cortex, which are related to walking tasks. Generally, the primary reasons for motion artifacts during walking tasks are dislocation of the optode and swaying of the optical cable [28]. Therefore, the first step in the proposed method is to detect the unbalanced optodes to find the most contaminated optode that has a motion artifact. The neighbor channels can be of two types: Those surrounding T-optodes and those surrounding R-optodes. In Figure 2b, the neighbor channels of CH 24 are CH 20, CH 19, and CH 15, which surround optode R6. CH 25, CH 21, and CH 16 are also neighbor channels of CH 20 and surround optode T7. If a motion artifact occurs at optode T7, the fNIRS signals of CH 25, CH 20, CH 21, and CH 16 will have substantial error, such as baseline drift and baseline jumping. However, the neighbor channels of CH 20 around optode R6 will not be affected by the motion artifact of optode T7; thus, the fNIRS signals in the channels around optode R6 will become unbalanced.

### 2.2. Contaminated Channel Identification Algoriothm Based on Entropy

**Figure 3:**
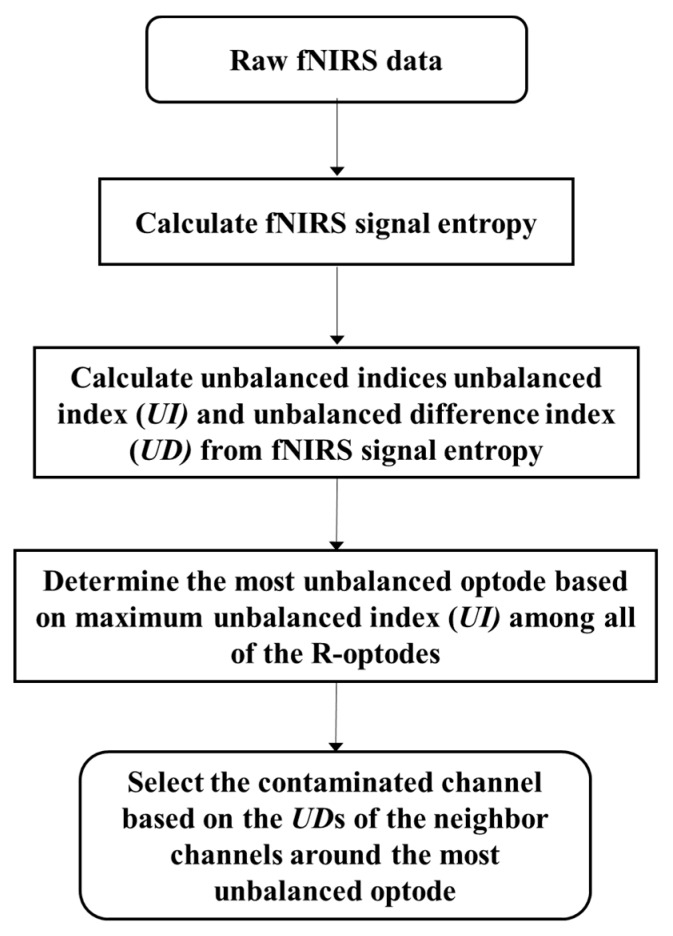
Contaminated channel identification flowchart.

The motion artifact correction method proposed in this paper involves selective processing for each channel affected by a motion artifact. Therefore, the channels requiring correction must be identified before signal correction is performed. These channels can be detected by observing the imbalance among neighbor channels. Figure 3 summarizes the process of contaminated optode and channel detection.

To detect the contaminated optode, signal entropy imbalance is used. Signal entropy has been utilized previously to extract the statistical features of signals [29]. Recently, signal entropy has been widely adopted in numerous fields, such as for signal noise reduction, time–frequency signal analysis, and feature classification [30,31,32]. The signal entropy can be calculated not only based on statistical features, but also by using time or frequency data [31], according to the following formula [29]:

(1) $$E=-\overset{N}{\underset{i=1}{\sum }}a\left(i\right)log_{2}\left|a\left(i\right)\right|.$$

Here, *a*(*i*) is the *i*th sample signal, *N* is the number of samples, and *i* is sample index. In fNIRS, the entropy of the HbO signal of each channel $E_{\mathrm{HbO}}^{\mathrm{ch}}$ and the mean entropy of the neighbor channels around an optode, except for channel *ch*, ${\bar{E}}_{\mathrm{HbO}}^{\mathrm{ex}\_\mathrm{ch}}$ can be respectively represented in terms of the time window *n* as follows:

(2) $$E_{\mathrm{HbO}}^{\mathrm{ch}}\left(n\right)=-\frac{1}{N}\sum y_{\mathrm{HbO}}^{\mathrm{ch}}\left(n-N/2:n+N/2\right)\cdot \log_{2}{|y}_{\mathrm{HbO}}^{\mathrm{ch}}\left(n-N/2:n+N/2\right)|,$$

(3) $${\bar{E}}_{HbO}^{\mathrm{ex}\_\mathrm{ch}}\left(n\right)=mean[E_{HbO}^{neigh\_ch1}\left(n\right),E_{HbO}^{neigh\_ch2},\ldots ]$$

Here, $y_{\mathrm{HbO}}^{\mathrm{ch}}$ is measured as HbO fNIRS at channel *ch*, *ch* is a specific channel (*ch* = 1, 2, …, 40), *Opt* is T- or R-optode number (*Opt* = T1, T2, …, T13, and R1, R2, …, R12), $E_{\mathrm{HbO}}^{\mathrm{ch}}$ is the signal entropy of fNIRS signal, and ${\bar{E}}_{\mathrm{HbO}}^{\mathrm{ex}\_\mathrm{ch}}$ is the mean entropy of the neighbor channels around the optode *Opt* apart from the channel *ch*. *N* was set to 20 samples (2 s) and the fNIRS signal entropy ($E_{\mathrm{HbO}}^{\mathrm{ch}}$) is calculated from 1 s before to after 1 s after *n*. To identify the contaminated optodes, the unbalanced index of the optode *UI^Opt^* and cross-correlation $\Gamma^{\mathrm{ch}}$ between $E_{\mathrm{HbO}}^{\mathrm{ch}}$ and ${\bar{E}}_{\mathrm{HbO}}^{\mathrm{ex}\_\mathrm{ch}}$ are employed, where each channel has two cross-correlation values since there are two optode types. These quantities can be computed as follows:

(4) $$\Gamma_{{\mathrm{Opt}}_{\mathrm{typ}}}^{\mathrm{ch}}=\sum_{i=1}^{N}E_{\mathrm{HbO}}^{\mathrm{ch}}\left(i\right)\;{\bar{E}}_{\mathrm{HbO}}^{\mathrm{ex}\_\mathrm{ch}}(i),$$

(5) $${\mathrm{UD}}^{\mathrm{ch}}=|\Gamma_{\mathrm{Opt}\_T}^{\mathrm{ch}}{-\Gamma }_{\mathrm{Opt}\_R}^{\mathrm{ch}}|,$$

(6) $${\mathrm{UI}}^{\mathrm{Opt}}=\mathrm{sum}({\mathrm{UD}}^{\mathrm{neigh}\_\mathrm{chs}}),$$

where *UD^ch^* is the unbalanced difference index at channel *ch*, which is coupled to an R-optode and a T-optode, and *Opt_Typ* indicates the T- and R-optode that share channel *ch* (e.g., for CH 20, *Opt_R* = R6 and *Opt_T* = T7). When the neighbor channels around optode *Opt* have different features and a low correlation, *UI^Opt^* is high. The contaminated optode can be identified using *UI^Opt^*, and the difference of the optode that has motion artifacts can be determined using *UD^ch^*.

### 2.3. Motion Artifact Correction Using BPNN

WT is a widely used tool that can provide a time-frequency representation of a signal. It is possible to resolve high-frequency components within a small time window of the signal [32]. Generally, WT is employed to decompose a signal by transforming a wavelet packet into time–frequency wavelet coefficients of multiple sub-bands [33,34]. In this study, WT decomposition was designed to represent the time–frequency form of the fNIRS signal using the Daubechies6 wavelet basis. For WT level *j*, the wavelet packet transform decomposes the fNIRS signal *y*(*n*) into 2*j* sub-bands corresponding to a set of wavelet coefficients *w_j,m_*(*k*):

(7) $$w_{j,m}(k)=\mathrm{WT}[y(n),j].$$

**Figure 4:**
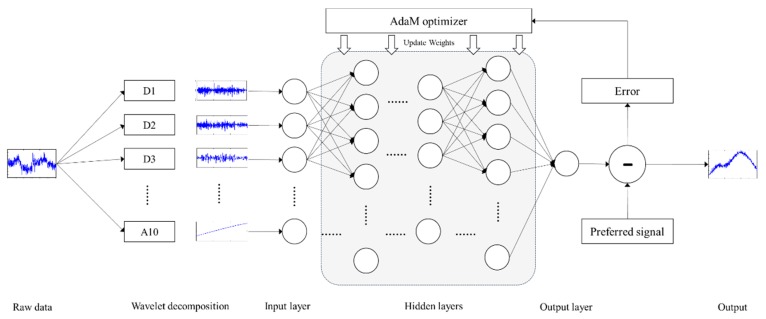
Structure of the proposed neural network.

The fNIRS signal is decomposed into 11 sub-bands with wavelet coefficients *w_j,m_*(*k*) by performing 10-level WT. Here, *w_j,m_*(*k*) is the *j*th level, *k*th wavelet coefficient of the *m*th sub-band in the WT, (*j* = 1, 2, …, 10; *m* = 1, 2, …, 11; and *k* = 1, 2, …, *N*/2*j*). The decomposed fNIRS signal obtained via WT has the time-frequency representation of an fNIRS signal in the time domain. Generally, as the baseline drift of fNIRS signal is observed in the low-frequency components, it can be removed using a high-pass filter. However, the baseline drift can be confused with brain activation and motion artifacts in some experimental environments such as one-block and short-time tasks. Moreover, baseline jumping, which is caused by rapid head movements, has spectral features very similar to those of BOLD signals related to brain activation. Motion artifacts have often not been removed effectively in such situations in previous studies. To overcome this issue, a technique involving machine training and restoration by using a BPNN is proposed in this paper. The BPNN structure applied in the proposed method is depicted in Figure 4, which shows that the proposed ANN consists of an input layer that receives wavelet sub-bands from the input data of the network, six hidden layers with more than 400 neurons, and an output layer with one neuron to obtain a linear regression output. ANNs are biologically inspired networks that have numerous applications in the field, such as in pattern recognition and classification.

In this method, the fNIRS signal, which has motion artifacts, is corrected by a BPNN. The BPNN is trained using the mean square error (MSE) function, which is commonly utilized to minimize the overall errors in a system. A BPNN generally includes a gradient descent search algorithm to get minimum MSE between the preferred and actual network outputs by adjusting the weights [22]. As the gradient descent optimization algorithm requires a substantial amount of time to achieve convergence, an AdaM optimizer [35] is employed in the BPNN in the proposed method to reduce the computing time. The decomposed fNIRS signal is applied to the input data, and each sub-band is entered into each input neuron. Multiple linear regression (LR) is based on the relationships between the dependent variable and explanatory variables. The most important assumption in this method is that the relationships between the dependent variable and the predictor variables are linear [23]. The LR equation can be used to forecast the desired value with the appropriate predictor variables. The network output and the weights of the hidden layers can be computed using multiple wavelet LR and the BPNN as follows [28]:

(8) $$X^{\mathrm{ch}}{(n)=\mathrm{mean}(y}_{\mathrm{HbO}}^{\mathrm{neigh}\_\mathrm{chs}}(n))$$

(9) $$Y^{\mathrm{ch}}=\left[\begin{array}{ccc} \omega_{1,1} & \cdots & \omega_{1,L}\\ \omega_{2,1} & \cdots & \omega_{2,L}\\ \vdots & \ddots & \vdots \\ \omega_{n,1} & \cdots & \omega_{n,L} \end{array}\right]\left[\begin{array}{c} D1\\ D2\\ \vdots \\ D10\\ A10 \end{array}\right].$$

*X^ch^* is the preferred fNIRS signal at channel *ch*, which is estimated from the mean fNIRS signals of the channels neighboring channel *ch*, apart from channel *ch* itself; and *ω_n,L_* are the network weights, which are optimized by AdaM optimization based on the MSE between *X^ch^* and the network output. *L* is length of the fNIRS signal samples.

## 3. Ex. Perimental Results

**Figure 5:**
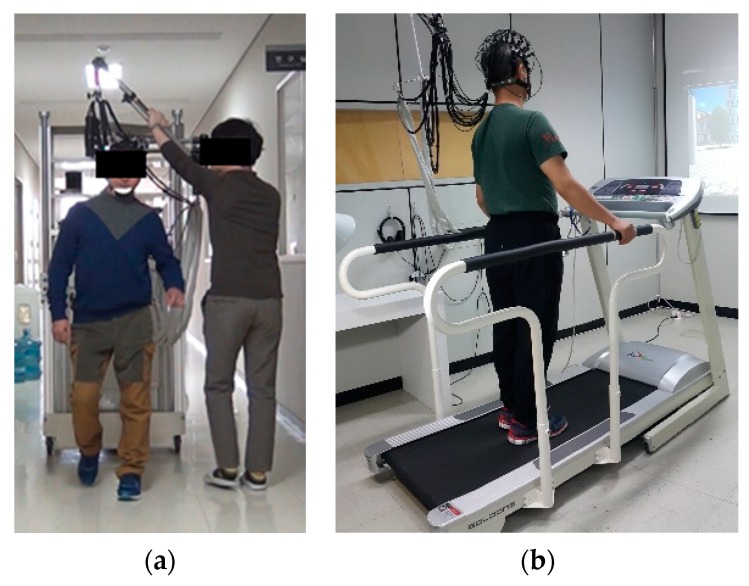
Actual experimental environment: (**a**) OG walking task; (**b**) TM walking task.

**Figure 6:**
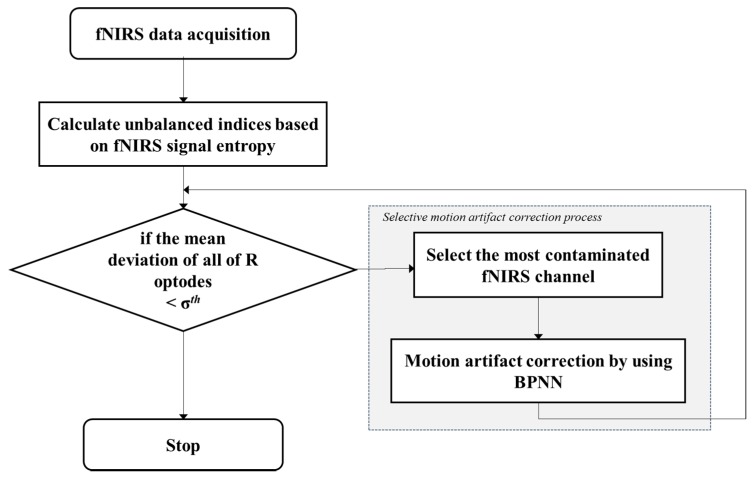
Flowchart of the proposed method.

For this experiment, six subjects (Two stroke patients and four healthy subjects, all male, and 34–57 ages) participated in this experiment, in which they performed normal walking motion both overground (OG) and on a treadmill (TM) in a block task design. The walking speed is 0.33 m/s, task and rest duration are both of 20 s, the block design is 20-20-20, and experimental duration is 30 min per day. Figure 5 shows the experimental environment, specifically, the OG walking task, TM walking task, and fNIRS cap. As can be seen in Figure 5a, the fNIRS system could be moved during the OG walking task by using a carrier. However, during the experiment, motion artifacts could occur for many reasons, such as head movements, swaying of the optic fibers, and optode issues. Figure 6 provides a flowchart of the proposed motion artifact correction method. Each step was performed according to the flowchart in this study.

**Figure 7:**
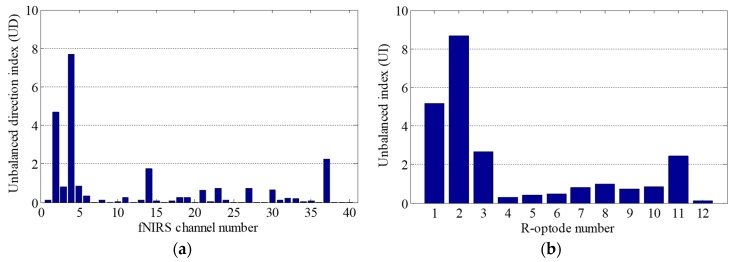
Contaminated optode identification: (**a**) *UD* for all of the fNIRS channels; (**b**) *UI* for all of the R-optodes.

Firstly, *UI* and *UD* were calculated based on the fNIRS signal entropy. Then, to correct the motion artifacts, the contaminated optodes were identified by using the *UI* values of the R-optodes. The *UI* values and fNIRS data obtained experimentally in the OG walking task are presented in Figure 7. Figure 7a shows *UD* for all of the fNIRS channels. Figure 7b demonstrates that the *UI* values of the R-optodes vary significantly. The *UI* of optode R2 is the highest, at 8.67, which indicates that optode R2 is the most contaminated optode. Thus, there were probably motion artifacts in the fNIRS signals near optode R2. The next step was to find the contaminated channel by using *UD*. The channels neighboring optode R2 were CH 3, CH 4, and CH 8. As the *UD* of CH 4 is the highest in Figure 7a, CH 4 was determined to be the unbalanced one and was then trained by using its neighbor channels (CH 3 and CH 8). Then, CH 4 was corrected using the deep neural network weights obtained by training the proposed BPNN algorithm. The T-optode neighbor channels of CH 4, which had features similar to those of CH 4, were also reconstructed using the network weights.

**Figure 8:**
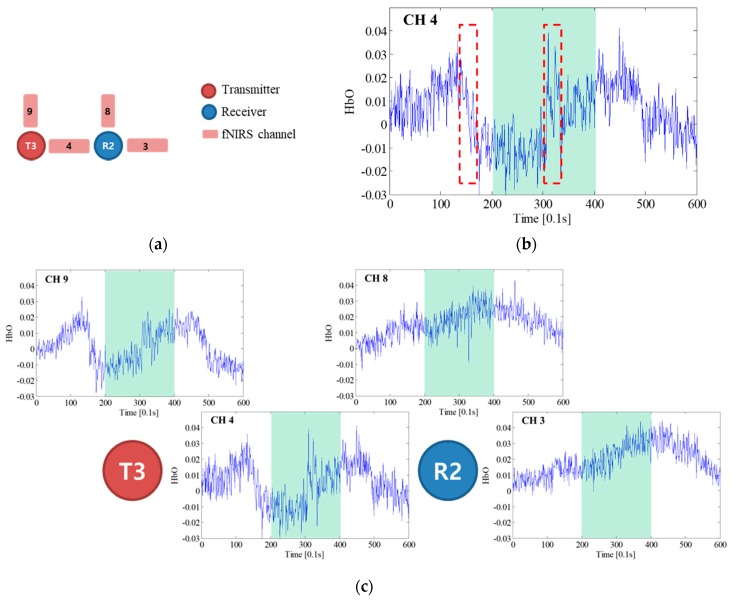
fNIRS signal near optode R2: (**a**) neighbor channels around optode R2; (**b**) fNIRS signal of CH 4; (**c**) fNIRS signals near optode R2 (the green background indicates the task block).

**Figure 9:**
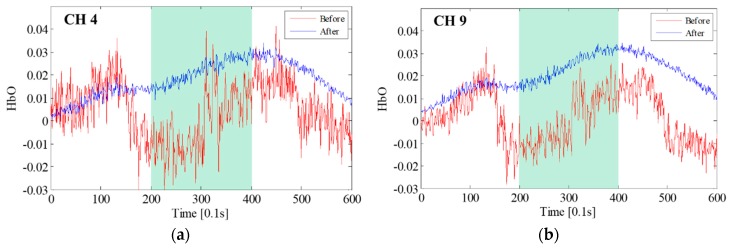
Deep neural network wavelet linear regression: (**a**) fNIRS signal correction of CH 4; (**b**) restoration and correction of CH 9 by using the obtained network weights (the green background indicates the task block).

The fNIRS signals near optode R2 and its neighbor channels, which were used to identify the most contaminated optode, are presented in Figure 8. Figure 8a shows the arrangement of the neighbor channels around optode R2 and the contaminated channels; Figure 8b is the fNIRS signal of CH 4, which was determined to be the most contaminated channel; and Figure 8c depicts the fNIRS signals measured near optode R2. Although the signals of neighbor channels have basically the same features, the trends of the CH 3 and CH 8 signals are quite different from those of the CH 4 signal, indicating that the artifact occurred at optode R2 or T3. Specifically, the fNIRS signals of CH 3 and CH 8 normally increase at the task block, while that of CH 4 decreases or varies dramatically, as can be seen in the red dashed boxes in Figure 8b. As HbO signals basically increase in the task block, it can be concluded that optode T3 had a motion artifact, which affected CH 4 and CH 9. By using Figure 8, the contaminated fNIRS channel identified based on the *UD* was confirmed to be correct. The next step was motion artifact correction using the proposed deep neural network wavelet regression. To correct the fNIRS signal of CH 4, the wavelet-decomposed signal of CH 4 was used as input for the network. The optimized network weights were then obtained by using the label signal acquired by averaging the signals of CH 3 and CH 8. The obtained weights were also applied to restore the neighbor channel of optode T3, which was CH 9. The graphical results obtained before and after motion artifact correction are presented in Figure 9a, where the fNIRS signal increases at the task block due to the motion artifact correction of CH 4 that was performed after conducting hundreds of thousands of training cycles with the proposed BPNN. Evidently, applying the proposed method not only removed the motion artifact components, but also reduced the measured noise present in the high-frequency components. It can also be seen that the hemodynamic fNIRS information was successfully retained. Figure 9b depicts the results obtained after restoration and correction of CH 9, the neighbor channel of CH 4 from optode T3. Because the fNIRS signals of CH 9 and CH 4 must have similar features, CH 9 should also have motion artifacts from optode T3, and the motion artifacts in CH 9 and CH 4 should have similar features.

Therefore, the motion artifacts could be corrected by using the previously obtained network weights. While Figure 9a was obtained after conducting more than 400,000 cycles with the BPNN, Figure 9b is the result of just 100 repetitions using the restored network weights from CH 4. These results demonstrate that satisfactory performance could be achieved by using minimal computing power. Finally, the corrected fNIRS signals were processed by low-pass denoising using HRF soothing. These processes were repeated for the other contaminated optode decisions and motion artifact corrections until the mean deviation of the *UI*s became less than *σ*th.

**Figure 10:**
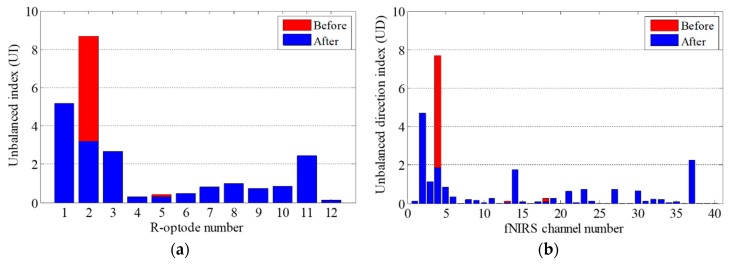
Changes of *UI* and *UD*: (**a**) *UI* for all of the R-optodes; (**b**) *UD* for all of the fNIRS channels.

The contaminated optode identification and motion artifact correction performance can be evaluated based on the *UD* and *UI* values calculated using the corrected fNIRS data. Figure 10 shows *UD* and *UI* for the original and corrected fNIRS data. As can be seen in Figure 10a, *UI* significantly decreased for optode R2, which was determined to be the most contaminated optode, and also decreased for optode R5, because it was located near optode R2. Figure 10b shows *UD* for all of the fNIRS channels. For CH 4, which was corrected using the proposed BPNN, *UD* exhibits an impressive decrease. Decreases are also evident in the *UD*s of CH 18 and CH 13, because they were the neighbor channels of CH 9. And these processes will be repeated for the next unbalanced optode.

**Figure 11:**
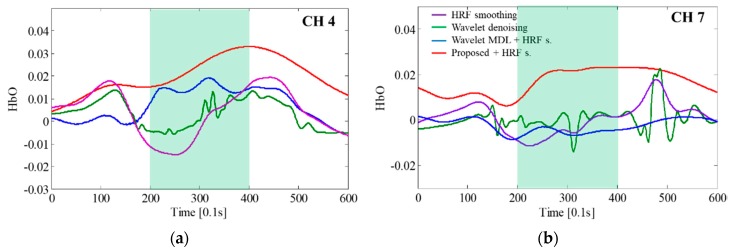
Comparison of the fNIRS signal correction results obtained using the proposed and conventional methods: (**a**) CH 4; (**b**) CH 7.

Figure 11 shows the motion artifact correction results obtained by using the proposed method and conventional methods. Figure 11a,b present the results for CH 4 and CH 7, respectively. Since the CH 7 is one of the ROI channel, the hemodymanic response will be present in CH 7. The results by using the proposed algorithm show the hemodynamic response in the task block of Figure 11a,b. However, the results of other algorithms cannot show an increase of hemodynamic response. Wavelet denoising and HRF smoothing evidently caused the fNIRS signals in the task block to decrease, indicating that they could not remove the motion artifacts. Meanwhile, the proposed method and wavelet MDL method caused the fNIRS signals to increase in the task block. Thus, the fNIRS signals were restored through the motion artifact correction. It should also be noted that the fNIRS signals, corrected by applying the proposed method, appear more natural than those obtained by employing the wavelet MDL approach. Specifically, the fNIRS signals corrected by using the wavelet MDL increase before the task block, which could have been caused by signal distortion or brain mapping errors.

**Figure 12:**
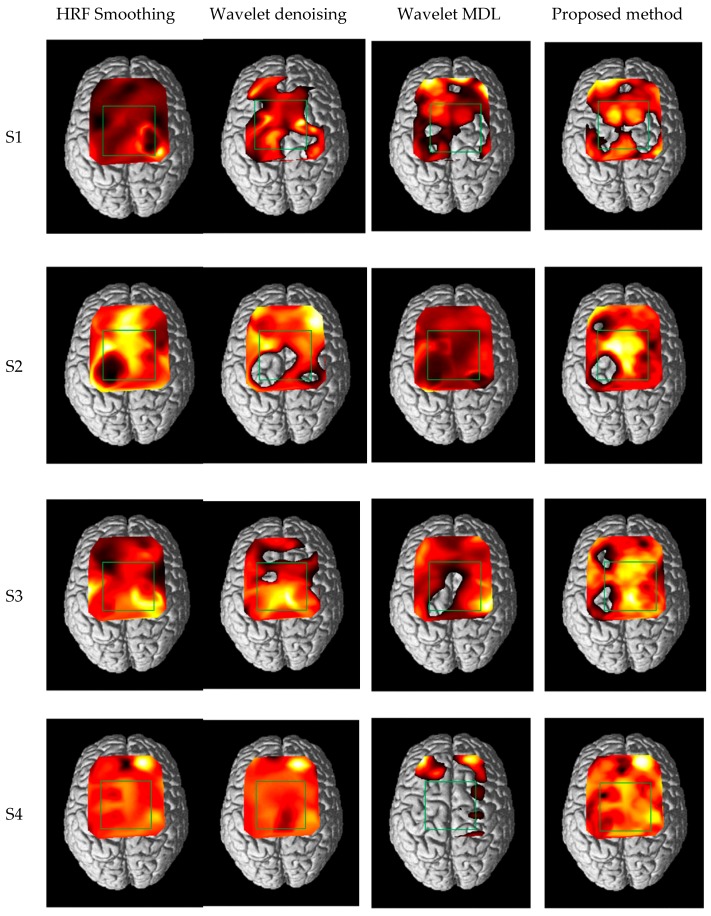

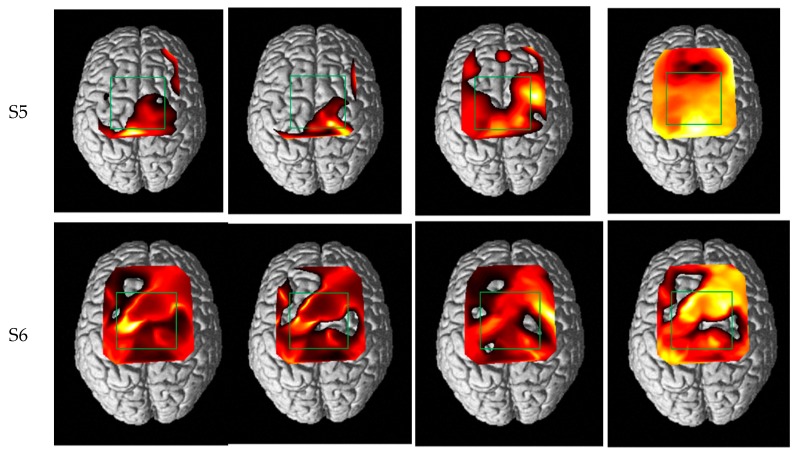
Brain activation map in comparing with conventional method and the proposed method (the green box: ROI).

Figure 12 shows one of the brain activation mapping results and the green box in Figure 12 presents the region of interest (ROI), which is related to the SMA (supplementary motor area) and M1 (primary motor cortex); in addition, ROI channels were determined (CH 7, 11, 12, 16, 20, 21, and 25). The ROI is decided based on previous fMRI, fNIRS and EEG studies [36,37,38,39]. As can be seen in Figure 12, the patterns of the brain activation are present in the similar region for each subject. Wavelet MDL tends to show quite different patterns because of the signal distortion, and HRF smoothing and wavelet denoising show similar activation maps. The brain activation of the proposed method shows stronger activation than other algorithms in ROI and it also presents symmetric activation. The result shows the proposed method has a feature-enhancing fNIRS signal and keep hemodynamic response of the brain cortex.

The proposed method was also evaluated more objectively by using a performance indicator. To evaluate the performances of the algorithms, the contrast-to-noise ratio (CNR), which evaluates the fNIRS signal quality by considering the statistical difference between the rest and the task block, has been widely applied. The CNR is computed as follows [40]:

(10) $$\mathrm{CNR}=\frac{\mathrm{mean}\left(\mathrm{task}\right)-\mathrm{mean}(\mathrm{rest})}{\sqrt{\mathrm{var}\left(\mathrm{task}\right)+\mathrm{var}(\mathrm{rest})}},$$

where *mean* is the mean value and *var* is the variation of the relevant part. As a hemodynamic response is evoked 3–5 s after starting a task block [41], the task and rest parts used for the CNR calculations were set to have a time delay of 4 s. If there is a large difference between the parts or a small variation in one part, the CNR is high. The higher the CNR, the better the fNIRS signal enhancement performance. Table 1 presents the detailed CNRs of the fNIRS data corrected by using the proposed and conventional methods. These values were statistically analyzed with six subjects, two experimental environments, and 1151 samples of fNIRS signal. To evaluate the performance of the proposed method, its performance was compared with those of three conventional methods, namely, the HRF smoothing [19], wavelet denoising, and wavelet MDL [11] methods. The wavelet MDL approach is one of the most widely used detrending algorithms for fNIRS. The wavelet denoising method is performed using the MATLAB toolbox [42] and involves setting a soft universal threshold and rescaling using a level-dependent noise level estimation [43,44,45]. Table 1 shows CNR results of all of the channels about each subject, and it also presents the results about corrected channels by proposed and conventional methods to evaluate fNIRS signal quality related to a walking task. And the CNR results on ROI channels (CH 7, 11, 12, 16, 20, 21, and 25) and on the corrected channels which are corrected by the proposed method are present on Table 2.

All of the methods show better CNRs in the TM case than in the OG case, because the gait task on the TM resulted in fewer motion artifacts. As the wavelet MDL method can cause too much signal distortion and diminution in certain situations, including those involving rapid changes such as the gait task, it shows poor performance for raw data which yields low CNRs. The wavelet denoising method provides constant though not very powerful CNR improvement. HRF smoothing exhibits performance similar to that of the proposed method overall. However, the proposed method shows higher CNRs and tends to be more robust when applied to raw data with a low CNR. Besides, in the corrected channels, the proposed method has the highest CNR in comparison to the other methods. Since ROI channels are directly related to a task, they have quite good quality and high CNR values in ROI channels. Therefore, most of the methods showed good performance in ROI channels and HRF smoothing showed slightly better result than the proposed method. However, the proposed method showed the best performance in most of the experimental environments. Thus, it was concluded that the proposed method provides better performance than the other methods based on the CNRs.

## 4. Conclusions

In this paper, a novel method of correcting motion artifacts in fNIRS signals was presented. In the proposed method, the contaminated optodes are identified by performing entropy cross-correlation with the neighboring fNIRS signals and the motion artifacts are corrected by using a BPNN. This method was designed based on the hemodynamic response of the cerebral cortex and spatial information of the brain in multi-measured fNIRS. The effectiveness of the proposed method was confirmed experimentally by analyzing the graphical results and CNRs obtained from gait tasks.

The proposed method may still have problems if it is applied to extremely poor fNIRS data with strong artifacts in most channels. Because the decision algorithm is based on the unbalancing of each optode or fNIRS channel, it cannot decide perfectly whether an fNIRS channel is normal or abnormal. Therefore, a detection algorithm for classifying abnormal fNIRS channels will need to be developed in future studies. In addition, the determination of the autonomous optimal ANN for linear regression, training scale, and optimization of the computing power should be further discussed.

The proposed method can be applied to the global detrending of fNIRS signals by using deep neural networks in task-oriented fNIRS experiments. It is also expected to be useful for post-processing, including neuro-cortical mapping and brain signal decoding for gait-based brain-computer interface.

## Figures and Tables

**Table 1:** Motion artifact correction results in comparing with the conventional and proposed methods.

		Raw Data		HRF Smoothing		Wavelet Denoising		Wavelet MDL		Proposed Method	
		OG	TM	OG	TM	OG	TM	OG	TM	OG	TM
Stroke patient subjects	S1	0.30	0.29	0.64	0.62	0.47	0.44	0.67	0.27	0.82	0.64
	S2	0.50	0.71	0.80	1.11	0.70	1.03	1.06	1.03	0.82	1.18
Healthy subjects	S3	0.85	1.06	1.19	1.58	0.97	1.27	0.53	1.28	1.25	1.62
	S4	0.05	0.18	0.18	0.35	0.01	0.12	0.06	0.17	0.22	0.36
	S5	0.20	0.25	0.38	0.51	0.34	0.80	0.09	0.39	0.42	0.54
	S6	1.04	1.00	1.46	1.52	1.14	1.22	1.43	1.18	1.52	1.51

**Table 2:** Motion artifact correction performance achieved using the conventional and proposed methods.

	HRF Smoothing	Wavelet Denoising	Wavelet MDL	Proposed Method
Corrected channels	0.49	0.36	−0.12	0.63
ROI channels	0.74	0.64	0.09	0.73
